# A Question of Food

**Published:** 1876-09

**Authors:** 


### A QUESTION OF FOOD.

Several inquiries have reached us respecting the wholesomeness of the cooked, compressed meats which are now largely sold all over the country. The papers in various localities report instances of serious illness attributed to these meats, and a child in New Bedford is said to have died from eating them. We have, therefore, considered it our duty to look into the subject, to learn the methods employed in preparing this now largely used food, and to get at the facts as nearly as possible, concerning the alleged poisoning, in order that we may advise our friends understandingly.

Several varities of meats are packed in a cooked state, but the leading article is beef, either fresh or slightly corned. Two kinds of packages are used, one of tin, the other of sheet-lead, which is called " tin foil." These last named packages are sold at lower prices, and have been largely employed by the poorer classes. We think this very unsafe food. If composed of good meat, originally, it speedily becomes tainted, because the air has never been exhausted from the packages. The means of protection from atmospheric action are altogether inadequate. Even if absolutely air-tight, we should anticipate lead-poisoning from the use of food thus wrapped in sheet-lead. If, therefore, serious illness follows the employment of this food, it should not occasion surprise, though it may well cause alarm.

Then their is another article of cooked meat in very general use, which is delicious in flavor, and very generally admired. It is put up in substantial tin cans. The cans are wedge-shaped to facilitate the removal of the contents unbroken. Two large establishments are engaged in packing meats in these cone-like cans, the leading one being known as "The Wilson Packing Company, of Chicago." Into their process we have looked with care, because their business vastly exceeds that of all other houses in this line. They slaughter about 200 choice beeves each day, cool the flesh, pickle it, steam it, remove the bones, force, by strong pressure, all that is edible into cans, exhaust the air, seal the cans, label them, and send them to the four quarters of the globe. If their product is wholesome and their work faithfully done, it must carry with it blessings innumerable ; if their meats are unwholesome or their work carelessly performed, it may be productive of vast injury.

Investigation satisfies us that the Wilson Packing Company exercise all possible care alike in the selection of their cattle and in the cooking and curing processes. They select well-conditioned beeves, the flesh of which is placed in a huge ice-chamber in order to withdraw the animal heat—a preservative measure. After this, it is pickled in the usual way. When lightly impregnated with salt it is cooked. This process occurs in wooden vats, through which pass steam pipes. Wood is preferred instead of the copper boilers used by others, because copper is likely to impregnate the food with poisonous metallic particles. When the meat is steamed to tenderness, it is removed from the bones and tendons, the pure meat is pressed into the wedge-shaped forms, and the cans are sealed in a hot state. Ordinarily, the contents of these can will keep a life-time. Now and then a can swells because of a failure on the part of the sealer to perfectly exclude the air, and this is a sign that the decomposition of the meat has generated a gas which has induced the expansion. When a can presents a bulged appearance it is immediately destroyed, as its odor speedily becomes terribly offensive.

The cans when properly sealed do not necessarily have to be kept in a cool place. When the top is removed the meat drops out in a molded form, like a pyramid of ice cream or jelly. The mottled meat is beautiful to the eye, fragrant to the sense of smell, and savory to the taste. But, like other meats, it will spoil in hot weather unless kept on ice. When tainted by incipient decomposition it can not be eaten with safety. Some wild birds and beasts are able to eat decayed meat without injury, but no human being ought to undertake it. Even the Indian tribes, who are accustomed to all sorts of unsavory foods, die by scores from blood-poisoning when compelled to subsist on spoiled meat.

No doubt the few cases of gastric disturbance which have followed the use of this meat, have either been occasioned by some other article of food partaken of at the same time, or have been induced by spoiled meat —that is, by keeping the contents of the can after opening it, until decomposition had set in. This would, of course, be a very dangerous experiment, and one which might sow the seeds of disease and death in the system.

We have been using the pressed corned beef of the Wilson Packing Company from time to time, for two or three years. We tested it chemically and microscopically and found it pure, clean, and wholesome, without other mixture than the flesh of beeves and saline matter. We found that the savory and nutritive juices of the meat—the most valuable portion—were carefully retained. So we have used the meat freely on our table and the result has been very satisfactory. It has proved a genuine delicacy as well as a substantial food, and is more economical than any meat bought of the butcher.

The cases of so-called poisoning by the Wilson Packing Company's meat, which we have sought information upon, have all finally been reduced to one of these conditions, namely, either some other brand of meat has caused the trouble, or the meat has undergone decomposition after being opened and before being eaten, or some substance with a known reputation for power to induce gastric disturbance—like cucumbers or lobsters—has been eaten at the same time. This fact, which we have authenticated, gives us the power to assert that the Wilson Packing Company's meats are as safe as any meats in the world, and that, like all other meats, they are not to be eaten after any taint or decomposition sets in.

The subject is important, because if we suffer from uncertainty as to the safety of our favorite foods, we are miserable indeed. In view of the great interests at stake, we have deemed it best to fortify our conclusions with the following testimony of some well known chemists :

[From Dr. S. Dana Hayes, State Assayer and Chemist, Boston, Mass.]

"I have repeatedly used the Wilson Packing Company's cooked corned beef within the past year, and have always found it to be good, and thoroughly preserved. Respectfully, S. DANA HAYES,
State Assayer and Chemist, Mass."

